# Supplementary material for: Identification of photoperiod-induced specific miRNAs in the adrenal glands of Sunite sheep (Ovis aries)
Source: Front Vet Sci. 2022 Jul 22;9:888207. doi: 10.3389/fvets.2022.888207 (PMC9354845; doi:10.3389/fvets.2022.888207)
Supplement: Supplementary file 1 [file Data_Sheet_1.ZIP › Supplementary file/miRDeep2 file.zip!/miRDeep2 file/Know_Structure/LP42A2/oar-mir-329a.pdf]

miRBase precursor : oar-mir-329a
Total read count : 66
oar-miR-329a-5p read count : 3
oar-miR-329a-3p read count : 62
remaining reads : 1

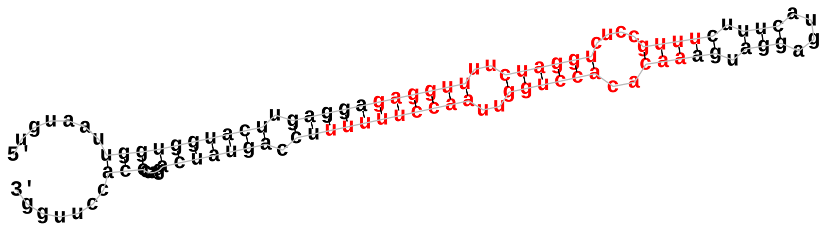

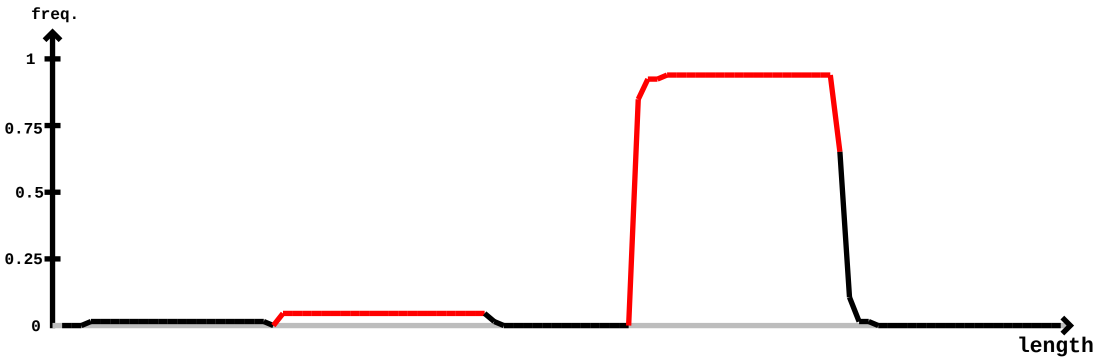

```
                            oar-miR-329a-5p
                                                                  oar-miR-329a-3p
5'- uguaauuggugguacuugaggagagguuuucuaggucuccguuucuuucaugaggaugaaacacaccugguuaaccuuuuuuccaguaucagaucccaccuugg   -3'    exp
    .......(((((((((.(((((((((((..((((((....(((((.(((....))).)))))..))))))..))))))))))).)))).......)))))....     reads     mm     sample
    ..uaauuggugguacuugagg...................................................................................     1         0      seq
    ......................gagguuuucuaggucuccguuu............................................................     2         0      seq
    ......................gagguuuucuaggucuccguuuc...........................................................     1         0      seq
    ...........................................................aacacaccugguuaaccuuuu........................     19        0      seq
    ...........................................................aacacaccugguuaaccuuuuu.......................     34        0      seq
    ...........................................................aacacaccugguuaaccuuuuuu......................     3         0      seq
    ............................................................acacaccugguuaaccuuuu........................     2         0      seq
    ............................................................acacaccugguuaaccuuuuuu......................     3         0      seq
    ..............................................................acaccugguuaaccuuuuuucc....................     1         0      seq
```
